# Radiation dose for 320‐row dose‐modulated dynamic coronary CT angiography

**DOI:** 10.1002/acm2.13390

**Published:** 2021-08-10

**Authors:** Yukako Izoe, Michinobu Nagao, Mei Tokai, Hiroyuki Hashimoto, Isao Tanaka, Koichi Chida

**Affiliations:** ^1^ Graduate School of Medicine, Health Sciences Division of Radiological Examination and Technology Tohoku University Sendai City Japan; ^2^ Department of Diagnostic imaging & Nuclear Medicine Tokyo Women’s Medical University Tokyo Japan; ^3^ Department of Radiological Service Tokyo Women’s Medical University Tokyo Japan

**Keywords:** 320‐row scanner, coronary artery disease, coronary CT angiography, radiation exposure

## Abstract

**Objectives:**

The area detector 320‐row CT scanner, which can cover the whole heart in one rotation, can aid in reducing radiation exposure during electrocardiography (ECG)‐gated coronary CT angiography (CCTA). Recently, researchers have proposed dose‐modulated dynamic CCTA with a 320‐row scanner for the detection of functional myocardial ischemia. In the present study, we compared and validated the radiation dose of this method with that of the standard CCTA method and the latest diagnostic reference levels (DRLs).

**Materials and Methods:**

The study included a total of 164 consecutive patients with suspected or known coronary artery disease (CAD) who underwent CCTA with a 320‐row scanner. The patients were randomly divided into dynamic and standard CCTA groups, and the CT dose index (CTDIvol) and dose length product (DLP) calculated by the CT system were compared between the two protocols and with the latest DRL.

**Results:**

Standard and dynamic CCTA scans were performed in 77 and 87 patients, respectively. CTDIvol was significantly higher for standard CCTA than for dynamic CCTA (41 ± 35 mGy vs. 22 ± 7 mGy, *p *= *0*.*0014*). DLP was also significantly higher for standard CCTA than for dynamic CCTA (864 ± 702 mGy × cm vs. 434 ± 106 mGy × cm, *p *< .*0001*). For standard scans, CTDIvol and DLP exceeded the 2020 DRL in Japan in 16% (12/77) and 17% (13/77) of cases, respectively. In contrast, rates for the dynamic scan were only 1% (1/87) for CTDIvol and 0% (0/87) for DLP.

**Conclusion:**

The dose of radiation exposure during dynamic CCTA with a 320‐row scanner does not exceed that of standard CCTA and is sufficient to meet the latest DRL. Thus, our results suggest that the method is safe from the perspective of radiation exposure.

## 1 INTRODUCTION

Japan is known to have the highest level of medical radiation exposure worldwide. It is estimated that approximately 60% of medical exposure occurs during CT examinations.^1^ Contributing to this is the widespread use of more than 10,000 CT scanners in Japan, which is the largest number in the world.^2^ Recent multi‐detector CT scanner advances in high‐speed and wide‐area scanning have been remarkable. CT examinations have doubled between 1996 and 2014, and approximately 30 million CT examinations are currently performed annually.^3^ An increase in the number of CT examinations could further increase medical radiation exposure among the population. The increased risk of carcinogenesis due to radiological examinations represents a serious public health concern.^4^ Thus, it is necessary to accurately investigate the actual extent of radiation exposure during radiological examinations. In 1990, the International Commission on Radiological Protection (ICRP) recommended the use of diagnostic reference levels (DRLs) for patient radiation exposure and image quality control.^5^ The DRL is a simple indicator to identify facilities where patient radiation exposure is greater than that of a typical facility.^6^ The DRL criteria for CT stipulated that the CT dose index (CTDIvol) and dose length product (DLP) should be used.^7^, ^8^ 

With the advent of a 64‐row CT scanner in 2004, it is now possible to perform noninvasive imaging of the coronary arteries.^9^ The method for diagnosing coronary artery stenosis has changed from the use of an invasive catheter to coronary CT angiography (CCTA). Furthermore, the 320‐row CT scanner, which has been in clinical use since 2007, can scan a range of 16 cm at once, enabling highly accurate coronary angiography within a shorter scan time.^10^ Previous studies comparing the exposure doses of 64‐row CT, second‐generation (128‐slice) dual‐source CT, and 320‐row CT have reported that predicting scan timing and adaptive iterative dose reduction 3D (AIDR 3D) has the effect of reducing the exposure dose in CCTA with a 320‐row scanner.^11^, ^12^

Functional coronary stenosis, rather than anatomic coronary stenosis, is key in determining whether to perform coronary interventions such as revascularization therapy.^13^ Recently, Kojima et al. proposed a new method for quantifying resting coronary flow estimated by 320‐row dynamic CCTA combined with a low radiation dose and boost scan.^14^, ^15^ While this may be a new noninvasive method for detecting functional coronary stenosis, it risks increasing radiation exposure. Therefore, we aim to verify and compare the dose of radiation exposure for 320‐row dynamic CCTA with that for standard CCTA and the latest CCTA diagnostic reference levels (DRLs) for Japan and Western countries.^16^, ^17^

## 2 METHODS

### 2.1 Study population

The study included a total of 186 consecutive patients with suspected or known CAD who underwent CCTA using a 320‐row CT scanner (Aquilion One Genesis Edition; Cannon Medical Systems Co., Tochigi, Japan) at our institution between May 2020 and November 2020. Of the 186 patients, 22 patients with a body weight of <40 kg or >80 kg were excluded. A total of 164 patients were finally included. Patients with a history of arrhythmia or previous treatment were scanned using the standard CCTA protocol, while the others were randomly divided into dynamic CCTA and standard CCTA protocols. Age, sex, height, and body weight were collected from the patients’ medical records. CTDIvol and DLP were collected from the dose recordings of the CT equipment.^8^ The concept of CTDI is a dose profile that is mountainous in the z‐axis direction, measured in a single scan with a detector that in long in the z‐axis direction. CTDIvol is the CTDI corrected for helical pitch. DLP is the line‐integrated dose, which is the total dose a patient receives in a single examination. The values calibrated using a 32‐cm‐diameter phantom for adult physical examination. All participants provided written informed consent for participation in the study, which was approved by the institutional ethics committee.

### 2.2 Dose‐modulated dynamic CCTA

**FIGURE 1:**
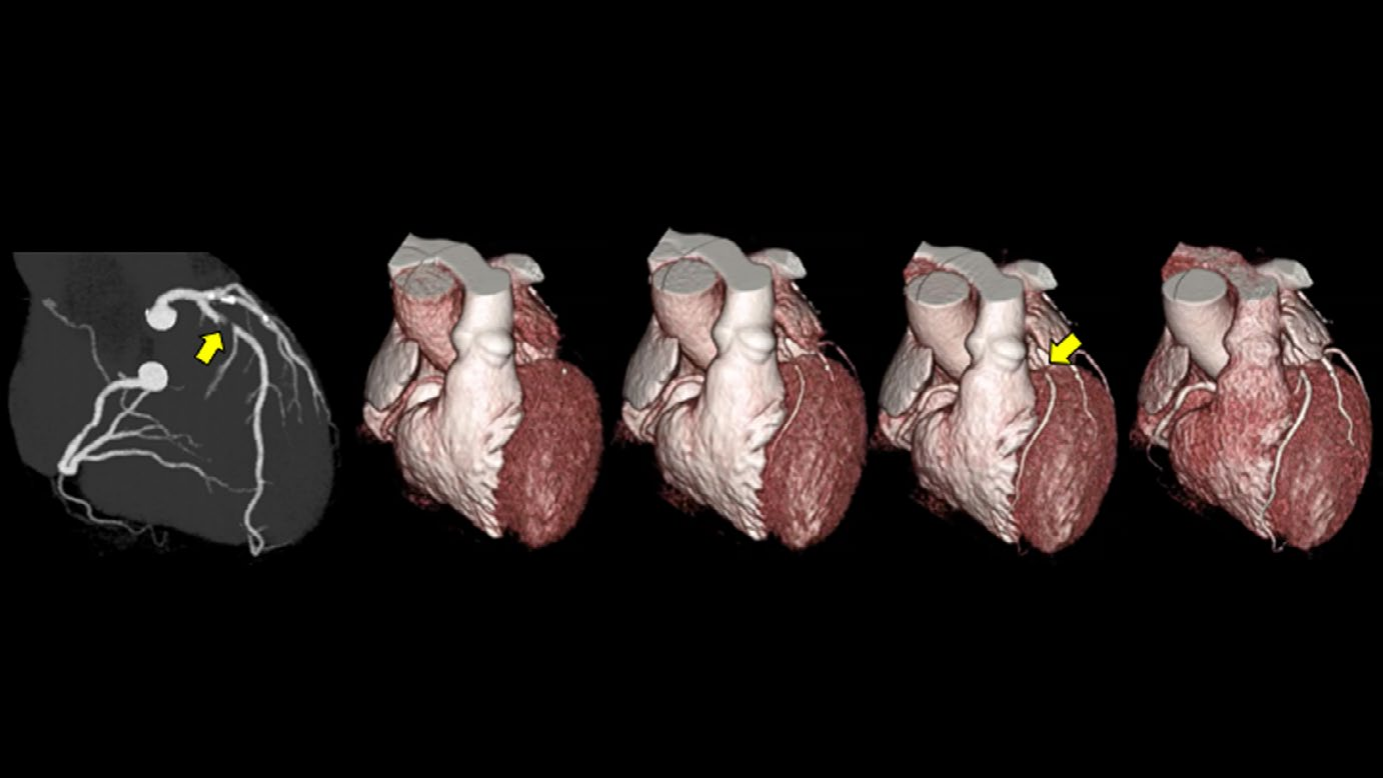
Angiographic view during the boost scan (left) and whole‐heart volume‐rendering images obtained from dynamic CCTA for a man in his 60s with severe stenosis at the proximal left anterior descending artery (arrows). Dynamic volume‐rendering images demonstrate the first pass of contrast medium from the heart cavities into their coronary arteries. Flow is traveling from the left to right over time. CCTA: coronary computed tomography angiography

This dynamic scan protocol was developed based on previous studies.^14^, ^15^ Intravenous or oral metoprolol (20 mg) was administered to patients with a heart rate of ≥65 beats/min. Immediately before image acquisition, all patients received sublingual nitroglycerine (0.2 mg). First, the test‐bolus examination was performed using non‐electrocardiography (ECG)‐gated axial scans at the ascending aorta to determine the optimal scan timing. Next, dynamic CCTA was continuously performed in mid‐diastole for 8–12 cardiac cycles with prospective ECG‐gating scans after a 10‐s injection of contrast medium (259 mg/kg, Iopamiron 370; Bayel Healthcare, Osaka, Japan). One scan of the dynamic CCTA was performed as a boost scan for standard CCTA at the peak phase of the ascending aorta (Figure 1). The acquisition parameters are presented in Table 1.

**TABLE 1:** Scan parameters for dynamic CCTA and standard CCTA

	Dynamic CCTA		Standard CCTA
	Dynamic scan	Boost scan	
Bean collimation (mm)	320 * 0.5		320 * 0.5
Tube voltage (kVp)	100		120
Tube current (mA)	80	AEC	AEC
Rotation time (s)	0.275		0.275
Exoposure phase (RR intercal %)	Fixed 70–80%		HR<65bpm, 70–80%; one beat
			HR>65bpm, 35–80%, two beat
Reconstruction	half		HR<65bpm, half; HR>65bpm, segment
Contrast material dose (mgI/kg)	259		259
Injection duration (s)	10		10
Iterative reconstruction	FIRST		FIRST

AEC: auto exposure control

### 2.3 Standard CCTA

Premedication and contrast protocols for standard CCTA were the same as those for dynamic CCTA. In this protocol, the region of interest in the ascending aorta was set, and a prep scan was performed to determine the timing of CCTA acquisition. If the heart rate was less than 65 bpm, the exposure phase was limited to 70–85% of the RR interval, and the image was scanned in one beat. If the heart rate was greater than 65 bpm, the exposure phase was expanded to 35–80% of the RR interval, and the image was scanned in two beats. The acquisition parameters are presented in Table 1.

### 2.4 Statistical analysis

Data are expressed as the mean ±standard deviation (SD) for continuous variables and as a number (percentage of total) for categorical variables. To examine differences in demographic data between the normal coronary CT group and the dynamic CT group, we used the unpaired Student's t‐test for continuous variables and the Chi‐square test for categorical variables. The differences in CTDIvol and DLP between the standard and dynamic CCTA groups were analyzed using the Mann–Whitney U‐test. Statistical significance was established using a two‐tailed *p*‐value <0.05. All statistical analyses were performed using the JMP statistical package (version 9.0; JMP, Inc., Cary, NC, USA).

## 3 RESULTS

The 164 patients (90 men [55%], 74 women [45%]) ranged in age from 21 to 89 years, with an average age of 62 years. Dynamic CCTA and standard CCTA were performed in 87 and 77 patients, respectively. Weight and heart rate were significantly higher in patients who underwent standard CCTA than in those who underwent dynamic CCTA. There were no significant differences in age or sex between the two groups (Table 2).

**TABLE 2:** Patient characteristics

	Dynamic CCTA	Standard CCTA	p value
	(N=87)	(N=77)	
Sex*1			
Male	45 (51.7%)	45 (58.4%)	0.3883
Age (years)^*2^	63.9 ± 12.9	59.4 ± 16.6	0.0552
Body weight (kg)^*2^	58.4 ± 8.1	62.5 ± 10.9	0.0076*
Heart rate (bpm)^*2^	58.0 ± 7.5	64.0 ± 10.9	<.0001*

^*1^ , Chi‐square test; ^*2^, Student's t test.

* Factors statistically significant (*p*<0.05); value are mean ±SD or number (percent).

**FIGURE 2:**
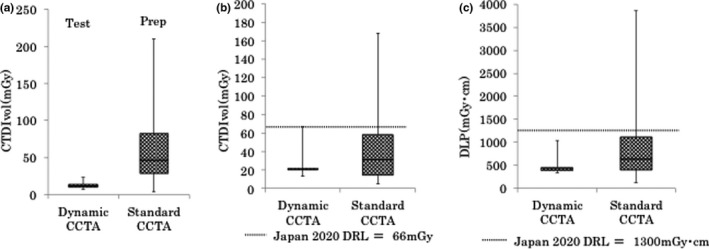
(a) Box‐and‐whisker plots of CTDIvol between the test scan in the dynamic protocol and prep scan in the standard protocol. The horizontal lines indicate the maximum and minimum, and the ends of the box (whiskers) and the line in the box indicate the upper and lower tertiles and median value, respectively. CTDIvol was significantly lower for the test scan than for the prep scan. (b) Box‐and‐whisker plots of CTDIvol between dynamic and standard CCTA. CTDIvol was significantly lower for dynamic CCTA than for standard CCTA. (c) Box‐and‐whisker plots of DLP between dynamic CCTA and standard CCTA. DLP was significantly lower for dynamic CCTA than for standard CCTA. CTDIvol: CT dose index; CCTA: coronary computed tomography angiography; DLP: dose length product

CTDIvol for the prep phase of standard CCTA was significantly higher than that for the test phase of dynamic CCTA (60 ± 44 mGy vs. 13 ± 3 mGy, *p *< .0001). CTDIvol for standard CCTA was significantly higher than that for dynamic CCTA (41 ± 35 mGy vs. 22 ± 7 mGy, *p *< .0001). The DLP for standard CCTA was significantly higher than that for dynamic CCTA (864 ± 702 mGy × cm vs. 434 ± 106 mGy × cm, *p *< .0001) (Figure 2).

The median CTDIvol for the prep scan and standard CCTA was 47 and 31 mGy, respectively. The median CTDIvol for the test scan and dynamic CCTA was 12 and 21 mGy, respectively. The median DLPs for standard and dynamic CCTA were 630 mGym × cm and 416 mGy × cm, respectively. For standard scans, CTDIvol and DLP exceeded the 2020 DRL for Japan in 16% (12/77) and 17% (13/77) of cases, respectively. In contrast, rates for the dynamic scan were only 1% (1/87) for CTDIvol and 0% (0/87) for DLP (Table 3). Results were nearly the same when compared to the 2019 DRL for Jordan. On the other hand, when compared to the 2018 Australian DRL, dynamic CCTA exceeded the DRL by 31% for CTDIvol and 100% for DLP (Table 3).

**TABLE 3:** Frequency of radiation dose above the DRL

	Dynamic CCTA(N=87)		Standard CCTA(N=77)	
	CTDIvol	DLP	CTDIvol	DLP
Japan DRL 2020	1/87 (1%)	0/87 (0%)	12/77 (16%)	13/77 (17%)
Jordan DRL 2019	2/87 (2%)	0/87 (0%)	24/77 (31%)	20/77 (26%)
Australia DRL 2018	27/87 (31%)	87/87 (100%)	50/77 (65%)	72/77 (94%)

Values are the number of cases over DRL / total number of cases (percent).

## 4 DISCUSSION

The present study investigated the doses of radiation exposure for standard and dynamic CCTA using a 320‐row scanner at a single clinical institution. The latter is a special scan technique that incorporates dose modulation to detect functional coronary stenosis. The median values of CTDIvol and DLP for both CCTA protocols were all below Japan's 2020 DRL. Indeed, for the dynamic protocol, CTDIvol and DLP exceeded the 2020 DRL in Japan in only 1% (1/87) and 0% (0/87) of cases, respectively. These rates were also lower than those for the standard CCTA protocol (Table 3). In 2014, totally 13,636 CT scanners were owned in Japan, of which approximately 400 were 320‐row scanners. The 2020 Japanese DRLs were created based on data from 180 facilities nationwide. Perhaps the 2020 Japanese DRLs largely reflect the dose of radiation exposure for 64‐row CT scanners. Our results indicate that 320‐row CT can be used to reduce the dose of radiation exposure for CCTA when compared with conventional 64‐row CT. When compared with the 2018 Australian DRL, dynamic CCTA exceeded the DRL by 31% for CTDIvol and 100% for DLP. Australia's 2018 DRL is lower than the DRL reported in most studies due to the implementation of dose‐saving technologies such as future ECG‐gating modes and iterative reconstruction algorithms.^16^ Although it represents a DRL under strict conditions that is not generalizable, it is necessary to continue optimizing Japan's DRL based on overseas situations.

Interestingly, CTDIvol and DLP were significantly lower for dynamic CCTA than for standard CCTA. In addition, dynamic CCTA had a narrower range for both CTDIvol and DLP than standard CCTA (Figure 2). These results are influenced by the fact that patients undergoing dynamic CCTA were lower in body weight than patients undergoing standard CCTA. Dynamic CCTA is heart rate‐independent, and prospective ECG gating is performed in all patients. On the other hand, in standard CCTA, if the heart rate is higher than 65 bpm, the exposure phase is expanded to 35–80% of the RR interval, and the image is scanned in two beats. In standard CCTA, the extension of the exposure phase or use of two‐beat scans is automatically selected depending on the heart rate. Furthermore, dynamic CCTA was performed at a low tube voltage of 100 keV, whereas standard CCTA was performed at 120 keV (Table 1). This difference in tube voltage affects the difference in the doses of radiation exposure between the two protocols. Our results demonstrate that dynamic CCTA can be performed with a low dose of radiation and with little individual variation.

The mean DLP for the original dynamic CCTA reported by Nagao et al.^15^ is approximately 630 mGy × cm, based on the effective radiation dose and the standard chest k‐factor of 0.014 mSv × mGy^−1^ cm^−1^. This DLP was higher than that for the current method (mean DLP: 434 mGy × cm). Recently, Kojima et al. reported a DLP of approximately 330 mGy × cm, estimated based on the standard chest k‐factor of 0.026 mSv ×mGy^−1^ cm^−1^ in a similar protocol.^14^ Their participants were mostly patients with significant coronary artery disease and were older than those in our study, with a mean age of 71 years. It is possible that differences in body size due to age differences affected the DLP. The present study is the first to compare the dose of radiation exposure between 320‐row CT, which is routinely performed, and the new method of dynamic CCTA with dose modulation. Furthermore, this is the first report comparing them with the latest Japan DRL, which is a stricter standard.^18^

We acknowledge the limitation that CTDIvol and DLP in this study are not dosimetric measurements. However, we measure CTDIvol and DLP one a year using a human phantom and a dosimeter. We have confirmed that these dosimetric measurements are consistent with the values estimated from the CT system. Furthermore, the values of CTDIvol and DLP estimated from 320‐row scanner are reported to be stable and less variable in large facilities such as ours.

## 5 CONCLUSION

The dose of radiation exposure during dynamic CCTA with a 320‐row scanner does not exceed that of standard CCTA and is sufficient to meet the latest DRL. Dynamic CCTA with a 320‐row scanner was able to reduce radiation exposure by 50% of the DLP compared to standard CCTA. If functional coronary stenosis can be detected using dynamic CCTA, our results suggest that the method is safe from the perspective of radiation exposure.

## ACKNOWLEDGMENT

This work was supported by the Japan Society for the Promotion of Science (JSPS) KAKENHI (19K08209).

## CONFLICT OF INTEREST

All authors declare no relationships with any companies whose products or services may be related to the subject matter of the article.

## AUTHOR CONTRIBUTIONS

All authors contributed to the study conception and design. Material preparation, and data collection and analysis were performed by Yukako Izoe, Michinobu Nagao, Mei Tokai, Hiroyuki Hashimoto, and Isao Tanaka. The first draft of the manuscript was written by Yukako Izoe, and all authors commented on previous versions of the manuscript. All authors read and approved the final manuscript.
